# Cross-border differences in public knowledge, awareness, behaviours and beliefs related to antibiotics and antimicrobial resistance across the island of Ireland

**DOI:** 10.1186/s12889-026-27024-w

**Published:** 2026-03-16

**Authors:** Caoimhe Shields, Emma Berry, Laura J. Sahm, Aoife Fleming, Chikondi C. Kandulu, Anne C. Moore, Gillian W. Shorter

**Affiliations:** 1 School of Psychology, Queen’s University Belfast, Belfast, Northern Ireland; 2 Pharmaceutical Care Research Group, School of Pharmacy, University College Cork, Cork, Ireland; 3 School of Biochemistry and Cell Biology, University College Cork, Cork, Ireland; 4 National Institute for Bioprocessing Research and Training, Dublin, Ireland; 5 SSPC Pharmaceutical Research Centre, University College Cork, Cork, Ireland; 6 School of Nursing and Midwifery, Trinity College Dublin, The University of Dublin, Dublin, Ireland; 7 TreAdd Research Group on Treatment and Addictions, Tampere University, Tampere, Finland; 8 Queen’s University Belfast, University Road, Belfast, BT7 1NN Northern Ireland

**Keywords:** Antimicrobial resistance, Public, Knowledge, Awareness, Behaviours, Beliefs, Antimicrobial stewardship, Behaviour change

## Abstract

**Background:**

Antimicrobial resistance (AMR) is predicted to be liable for 10 million annual deaths worldwide by 2050, driven significantly by public cognitions and behaviours. Given the frequent social and economic interactions between people from Northern Ireland (NI) and Ireland (IRL), there is potential for cross-border spread of antibiotic-resistant bacteria. Little research compares public knowledge, awareness, beliefs and behaviours across the island of Ireland. This study aimed to address this gap in a post-COVID-19 era to inform targeted interventions.

**Methods:**

A cross-sectional, nationally representative online survey with adults in NI and IRL assessed public knowledge, awareness, behaviours and beliefs related to antibiotics and AMR. Questions were taken from the World Health Organization (WHO) multi-country public awareness survey and four relating to ESKAPE pathogens were derived from literature. Statistical analyses of difference compared results between NI and IRL.

**Results:**

Among 811 respondents, 415 (51.2%) were from NI and 396 (48.8%) were from IRL. Those from NI showed better knowledge and understanding across most topics compared to those from IRL. However, effect sizes were small. Total knowledge of appropriate antibiotic use and antibiotic resistance was moderate in both countries. Nearly two fifths (37.9%) in both countries incorrectly identified ‘cold and flu’ as treatable with antibiotics. Awareness of AMR-related terms was consistent across countries and lowest for ‘ESKAPE pathogens’(11%), and ‘AMR’ (21.5%). The media (41.2%) and a doctor or nurse (27.1%) were the most frequent sources of awareness. Antibiotic use behaviours were consistent across countries, with over half (57%) having taken them within the last year. More respondents in IRL reported that there is not much they can do to stop antibiotic resistance (*U* = 74747.50, *p* = 0.02, *r* = 0.08).

**Conclusions:**

Knowledge, awareness, beliefs and behaviours around AMR and antibiotics are broadly consistent across the island of Ireland. Community-based initiatives could be used in both countries to educate the public on AMR and appropriate antibiotic use. Campaigns should refrain from using acronyms and employ multi-channel strategies to foster shared responsibility and encourage positive AMR-related behaviours across the island of Ireland.

**Supplementary Information:**

The online version contains supplementary material available at 10.1186/s12889-026-27024-w.

## Background

The World Health Organization (WHO) recognise antimicrobial resistance (AMR) as a major threat to public health and healthcare systems [1, 2]. Antibiotics which fail to kill bacteria lead to difficulties with infection prevention and control [3], increased burden on healthcare systems [4], and higher rates of morbidity and mortality [5]. Examples of bacteria which are highly resistant to antibiotics are a group known collectively as ESKAPE (E=*Enterococcus faecium*, S=*Staphylococcus aureus*, K=*Klebsiella pneumoniae*, A=*Acinetobacter baumannii*, P=*Pseudomonas aeruginosa*, and E=*Enterobacter* species) pathogens, which are mostly responsible for nosocomial (hospital acquired) infections [6]. 

Human cognitions like poor knowledge, lack of awareness, and misconceptions within beliefs have been described internationally as impeding the tackling of AMR [7–10]. The same has been said for human behaviours like high rates of inappropriate antimicrobial prescription and use [11, 12] and poor hygiene practices [6]. The effectiveness of infection prevention measures depends on behavioural insights [13], of which have been described as critical in the context of AMR [14]. Therefore, understanding these factors and their implications is fundamental in curbing the predicted AMR attributable death toll of 10 million annually worldwide by 2050 [15]. Much of the concern around AMR focuses on low-income countries due to poor sanitation and lack of access to clean water and healthcare [16–18]. However, AMR remains a concern in high-income countries for reasons such as high rates of antimicrobial consumption [19]. 

Northern Ireland (NI) and Ireland (IRL) (collectively termed here as the island of Ireland) face this problem. NI had the highest rate of total antibiotic consumption compared to England, Scotland, or Wales in 2021–2022; nearly double that of England (30.42 compared to 15.90 defined daily doses (DDD) per 1000 habitants per day) [20, 21]. Antibiotic consumption in IRL in 2023 was 22.4 DDD, which was high compared to other European Union (EU)/European Economic Area (EEA) countries (range of 9.57 to 28.52 DDD) [22]. IRL have targeted to reduce AMR-related deaths by 10% to 1,550 from 2019 figures by 2030, however trends suggest this could be up to 2,020 [23]. While directly comparable data is unavailable for NI, several bacterium in NI have shown increases in the proportion of isolates displaying multi-drug resistance between 2019 and 2022 (e.g., Pseudomonas spp. (2.4% to 16.1%). In addition, rates of Pseudomonas found in the water outlets of a NI hospital in 2024 resulted in the delay of its opening [24]. These high rates of antibiotic consumption and public health implications highlight AMR is a concern across the island of Ireland. 

Global research has shown AMR bacteria spread more frequently across countries and territories with increases in cross-trade and travel [25]. NI and IRL share an international border with no visa requirements for cross-border travel, permitting free movement and frequent social and commercial interactions. Research has found differences in the prevalence of resistant bacteria in four local authority areas (LAAs) in Ireland [26] and that AMR prevalence differs across 29 European countries (including IRL and the UK/NI), with subnational variance explaining approximately 38% of the total [27]. While the subnational variance in NI and IRL specifically has not been reported, these findings emphasise the importance of exploring and comparing national and subnational contexts and possible drivers of AMR influencers. NI and IRL have different healthcare systems and approaches to improving health literacy. Access to General Practitioner (GP) appointments and medication is free to all individuals under the National Health Service (NHS) in NI [28]. Depending on healthcare coverage (e.g., being eligible for a medical card or GP visit card) [29], individuals in IRL can access these either free of charge, at a capped price, or full price [30]. Multiple educational and community-based initiatives are delivered in NI; since 2005, to relieve workload pressures on GPs. These include the Pharmacy First Service [31] which aims to encourage the public to consult a community pharmacist first rather than the GP for common conditions, e.g., sore throat, diarrhoea, acne. In 2023, IRL established the Expert Taskforce to Support the Expansion of the Role of Pharmacy for the same effect, however it is not yet in operation [32]. Community-based initiatives like the Building the Community-Pharmacy Partnership Programme (BCPP) in NI integrates community pharmacies and community organisations to reduce health inequalities, including barriers to accessing healthcare, and improve health literacy [33]. No similar community initiatives are delivered in IRL. 

Awareness raising campaigns across the island of Ireland share common objectives and messages. The UK/NI’s ‘Keep Antibiotics Working’ [34] and IRL’s ‘RESIST’ [35] multi-channel social marketing and communications campaigns aim to raise awareness about AMR and the dangers of misusing antibiotics. The campaigns are promoted in community pharmacies, healthcare settings, and via broadcast advertising (television, social media platforms, radio and newspaper). Differences in healthcare provision and educational initiatives may contribute to variations in public knowledge, awareness, behaviours and beliefs regarding AMR, highlighting the importance of gaining country-specific insights. The application of behavioural science to improve public knowledge and awareness of AMR is a shared objective in NI and IRL National Action Plans (NAPs) [36, 37]. 

Available research on public cognition and behaviours related to antibiotics and AMR across the island of Ireland is country specific. Northern Irish research is limited and data is ten years old [38]. Recent IRL public research uses data just before and after the Coronavirus Disease 2019 (COVID-19) pandemic [9, 39, 40]. However, research suggests public knowledge, awareness, beliefs and behaviours around antimicrobials, infection and disease have changed since the COVID-19 pandemic [41, 42]. An up-to-date cross-border comparison of these factors is warranted to address this literature gap. This study aimed to explore cross-border differences in public knowledge, awareness, beliefs and behaviours related to antibiotic use and AMR across the island of Ireland. These latest behavioural insights can be used to inform public engagement strategies and tailor public health strategies which aim to improve antimicrobial stewardship (AMS) and population-based protection against AMR.

## Methods

### Study design, setting and participants

A mixed-methods cross-sectional survey was distributed online facilitated by Qualtrics research panel service in September 2024. To reduce selection bias, we asked Qualtrics to recruit a nationally representative sample based on the most recent NI and IRL national Census data on sex [43, 44]. Qualtrics recruited the participant pool through panel providers, who recruit via emails or portals. Cochran’s sample size formula [45] was used (95% confidence level and a margin of error of ± 5%). With this, the aim was to recruit 772 participants (386 in each country), therefore the quota was set at 800 to account for potential unusable responses. The survey took 10–15 min to complete. Adults living in NI or IRL were eligible to take part. Qualtrics advertised the study to those eligible within their participant panels. The target quota was reached within sixteen days, at which point the survey was closed. The exact reward rate for participation was allocated by Qualtrics (forms included SkyMiles, cash/gift cards, or retail outlet points).

### Measures

The survey (see Supplementary material 1) comprised six sections of 18 closed questions and 2 open-ended questions. Sections 1–4 were taken from the WHO antibiotic resistance multi-country public awareness survey [10]. Section 5 questions were informed by (a) existing literature [46] and (b) the WHO survey [10] by modifying the topic of its questions to focus on ESKAPE pathogens while maintaining the original question structures. These were not formally piloted before data collection. All other questions from the original WHO survey were not altered to preserve the psychometric properties and validity of the scale. Demographic data was collected using 14 questions. Eight were from the WHO [10]. Five were from EuroQol’s EQ-5D-3 L (EuroQol-5 Dimensions-3 Levels) standardised measure of health status [47]. The EQ-5D-3 L produces a health rating across five categories (mobility, self-care, usual activities, pain/discomfort, anxiety/depression), each measured on a 3-level scale (1 = no problems, 2 = some problems, 3 = extreme problems). Overall scores were calculated using Dolan (1997)’s UK coefficients [48]. The EuroQol Visual Analogue Scale (EQ VAS), part of the EQ-5D-3 L, was also used which rates overall health on a visual scale (0 = worst health you can imagine, 100 = best health you can imagine). The final measure was a yes/no question on whether the participant worked in any of the following sectors which could potentially be related to AMR; healthcare, public health, veterinary medicine, pharmaceutical industry, agriculture, environmental science, policy and government, research and academia. The following measures were used but not analysed in this study due to lack of relevance to the research question; measures of vaccine hesitancy [49] (Sect. 6), and two open-ended questions about the survey topics. Knowledge and awareness were measured using true/false or yes/no questions, or questions which required identifying one correct answer from multiple options. Behaviours were measured by the identification of occurrence of actions. Source-related questions were assessed from selection from a provided list. Beliefs were measured using a 5-point Likert scale (1 = strongly disagree, 5 = strongly agree).

### Statistical analysis

Data was exported from Qualtrics into IBM^®^ SPSS^®^ (Version 30) for analysis. Some demographic questions were optional, which was reflected in the response rates for each question. Eight of the main survey questions were asked conditionally if respondents answered ‘Yes’ to the related previous question which is reflected in the response rates. Therefore, missing values in these variables were considered as not applicable rather than missing. All remaining main survey questions were configured as forced response within Qualtrics, therefore there was no missing data to handle. Correct knowledge answers were based on what was deemed correct in the original measures, and for ESKAPE pathogen knowledge questions, from what is known about these pathogens from the literature. Correct answers were re-coded as a score of 1 and incorrect responses were grouped and coded as 0. Four sum variables were created from these to give each participant an overall score of topic knowledge, with higher scores denoting higher knowledge. Prevalence of sources of awareness and behaviours, and beliefs were analysed using descriptive statistics. Cross-border differences were calculated using Pearson chi-square tests of independence (categorical data), Mann-Whitney U tests (ordinal data), and independent t-tests (total scores data). Statistical significance was defined as p < 0.05.

## Results

### Sample demographics

A total of 811 responses were collected; 415 (51.2%) from NI and 396 (48.8%) from IRL (see Table 1). Of these, 407 (50.2%) were female, 398 (49.1%) were male, and five (0.6%) participants were self-described. Participant ages ranged from 18 to 89 years, with a mean age of 43.01 years and median age of 41 years (IQR = 32–53). Over one quarter of the sample were aged between 35 and 44 years (26.0%), with more in this age group in IRL (*n* = 118, 29.8%) compared to NI (*n* = 93, 22.4%) The most frequent educational level achieved was a Bachelor’s degree (*n* = 252, 31.3%), with more in NI having attained this compared to IRL (*n* = 114, 29.1%; *n* = 138, 33.4%). Most identified as white (*n* = 697, 86.3%). Most were married/in a domestic partnership and had at least 1 child under 16 (*n* = 260, 32.8%), more so those from IRL (*n* = 139, 35.9%) compared to those from NI (*n* = 121, 29.9%). Just under a quarter (*n* = 200, 24.7%) worked in a sector related to AMR. Total EQ-5D-3L average health index scores (*Mean*,* Standard Deviation*) were relatively poor (0.3, 0.14), ranged from − 0.06 to 0.87, and were similar in both countries (*t*(809) = 1.65, *p* = 0.10, *d* = 0.12). Those from IRL had a slightly higher average EQ-VAS perceived health score on the day of survey completion (74.60, 18.76) than those from NI (70.79, 22.44), (*t*(809)=-2.61, *p* = 0.009, *d*=-0.18).

**Table 1:** Comparison of IRL and NI survey respondents’ demographic characteristics (*n* = 811)

Characteristics	Valid n*	Mean (Standard Deviation) or *n* (%)		
		Total number (%)	Number in IRL (%)	Number in NI (%)
Gender	810			
Female		407 (50.2%)	200 (50.5%)	207 (50.0%)
Male		398 (49.1%)	194 (49.0%)	204 (49.3%)
Self-described		5 (0.6%)	2 (0.5%)	3 (0.7%)
Age (in years)	811			
18–24		82 (10.1%)	41 (10.4%)	41 (9.9%)
25–34		173 (21.3%)	81 (20.5%)	92 (22.2%)
35–44		211 (26.0%)	118 (29.8%)	93 (22.4%)
44–54		166 (20.5%)	83 (21.0%)	83 (20.0%)
55–64		101 (12.5%)	46 (11.6%)	55 (13.3%)
65+		78 (9.6%)	27 (6.8%)	51 (12.3%)
Urbanisation	811			
Rural		207 (25.5%)	103 (26.0%)	104 (25.1%)
Suburban		330 (40.7%)	163 (41.2%)	167 (40.2%)
Urban		274 (33.8%)	130 (32.8%)	144 (34.7%)
Highest level of education attained	805			
No qualifications		22 (2.7%)	8 (2.0%)	14 (3.4%)
Essential skills/Level 3 Junior Cert		22 (2.7%)	16 (4.1%)	6 (1.5%)
GCSE/Level 4 Leaving Cert or similar		106 (13.2%)	39 (9.9%)	67 (16.2%)
AS/A Level/Level 5 Leaving Cert or similar		158 (19.6%)	86 (21.9%)	72 (17.4%)
Technical qualification		114 (14.2%)	64 (16.3%)	50 (12.1%)
Bachelor’s degree		252 (31.3%)	114 (29.1%)	138 (33.4%)
Master’s degree		119 (14.8%)	60 (15.3%)	59 (14.3%)
Doctorate degree		12 (1.5%)	5 (1.3%)	7 (1.7%)
Total household income	780			
Less than £/€15,000		87 (11.2%)	31 (8.2%)	56 (14.0%)
£/€15,000 - £/€29,999		185 (23.7%)	89 (23.4%)	96 (24.0%)
£/€30,000 - £/€44,999		168 (21.5%)	72 (18.9%)	96 (24.0%)
£/€45,000 - £/€59,999		137 (17.6%)	76 (20.0%)	61 (15.3%)
£/€60,000 or more		203 (26.0%)	112 (29.5%)	91 (22.8%)
Ethnicity	808			
Asian Other		8 (1.0%)	6 (1.5%)	2 (0.5%)
Bangladeshi		2 (0.2%)	2 (0.5%)	-
Black African		34 (4.2%)	23 (5.8%)	11 (2.7%)
Black Caribbean		1 (0.1%)	1 (0.3%)	-
Black Other		5 (0.6%)	3 (0.8%)	2 (0.5%)
Chinese		4 (0.5%)	1 (0.3%)	3 (0.7%)
Indian		9 (1.1%)	6 (1.5%)	3 (0.7%)
Mixed ethnic group		15 (1.9%)	8 (2.0%)	7 (1.7%)
Other ethnic group		5 (0.6%)	2 (0.5%)	3 (0.7%)
Pakistani		9 (1.1%)	6 (1.5%)	3 (0.7%)
White		697 (86.3%)	323 (81.8%)	374 (90.6%)
White Irish Traveller		2 (0.2%)	2 (0.5%)	-
White Other		14 (1.7%)	9 (2.3%)	5 (1.2%)
White Roma		3 (0.4%)	3 (0.8%)	-
Household composition	792			
1 single adult only		156 (19.7%)	65 (16.8%)	91 (22.5%)
1 single adult and at least 1 child under 16		52 (6.6%)	28 (7.2%)	24 (5.9%)
Married / domestic partnership – adults only		174 (22.0%)	75 (19.4%)	99 (24.4%)
Married / domestic partnership and at least 1 child under 16		260 (32.8%)	139 (35.9%)	121 (29.9%)
Multiple adults aged 16 + only		116 (14.6%)	62 (16.0%)	121 (29.9%)
Multiple adults aged 16 + and at least one child under 16		34 (4.3%)	18 (4.7%)	16 (4.0%)
Work sector related to AMR	811			
Yes		200 (24.7%)	108 (27.3%)	92 (22.2%)
No		611 (75.3%)	288 (72.7%)	323 (77.8%)
EQ-5D-3 L scores by dimension and level	811			
*Mobility*				
Level 1. No problems in walking about		678 (83.6%)	345 (87.1%)	333 (80.2%)
Level 2. Some problems in walking about		127 (15.7%)	50 (12.6%)	77 (18.6%)
Level 3. Confined to bed		6 (0.7%)	1 (0.3%)	5 (1.2%)
*Self-care*				
Level 1. No problems with self-care		722 (89.0%)	376 (94.9%)	346 (83.4%)
Level 2. Some problems washing or dressing myself		84 (10.4%)	19 (4.8%)	65 (15.7%)
Level 3. Unable to wash or dress myself		5 (0.6%)	1 (0.3%)	4 (1.0%)
*Usual activities*				
Level 1. No problems with performing my usual activities		631 (77.8%)	331 (83.6%)	300 (72.3%)
Level 2. Some problems with performing my usual activities		162 (20.0%)	62 (15.7%)	100 (24.1%)
Level 3. Unable to perform my usual activities		18 (2.2%)	3 (0.8%)	15 (3.6%)
*Pain/discomfort*				
Level 1. No pain or discomfort		487 (60.0%)	263 (66.4%)	224 (54.0%)
Level 2. Moderate pain or discomfort		281 (34.6%)	120 (30.3%)	161 (38.8%)
Level 3. Extreme pain or discomfort		43 (5.3%)	13 (3.3%)	30 (7.2%)
*Anxiety/depression*				
Level 1. Not anxious or depressed		447 (55.1%)	231 (58.3%)	216 (52.0%)
Level 2. Moderately anxious or depressed		299 (36.9%)	145 (36.6%)	154 (37.1%)
Level 3. Extremely anxious or depressed		65 (8.0%)	20 (5.1%)	45 (10.8%)
EQ-5D-3 L health state index score		0.3 (0.14)	0.29 (0.13)	0.31 (0.14)
EQ-5D-3 L VAS perceived health score	811	72.65 (20.80)	74.60 (18.76)	70.79 (22.44)

*Some demographic questions were optional, therefore *n* ≠ 811 accounts for missing data

### Knowledge of appropriate antibiotic use

**Fig. 1:**
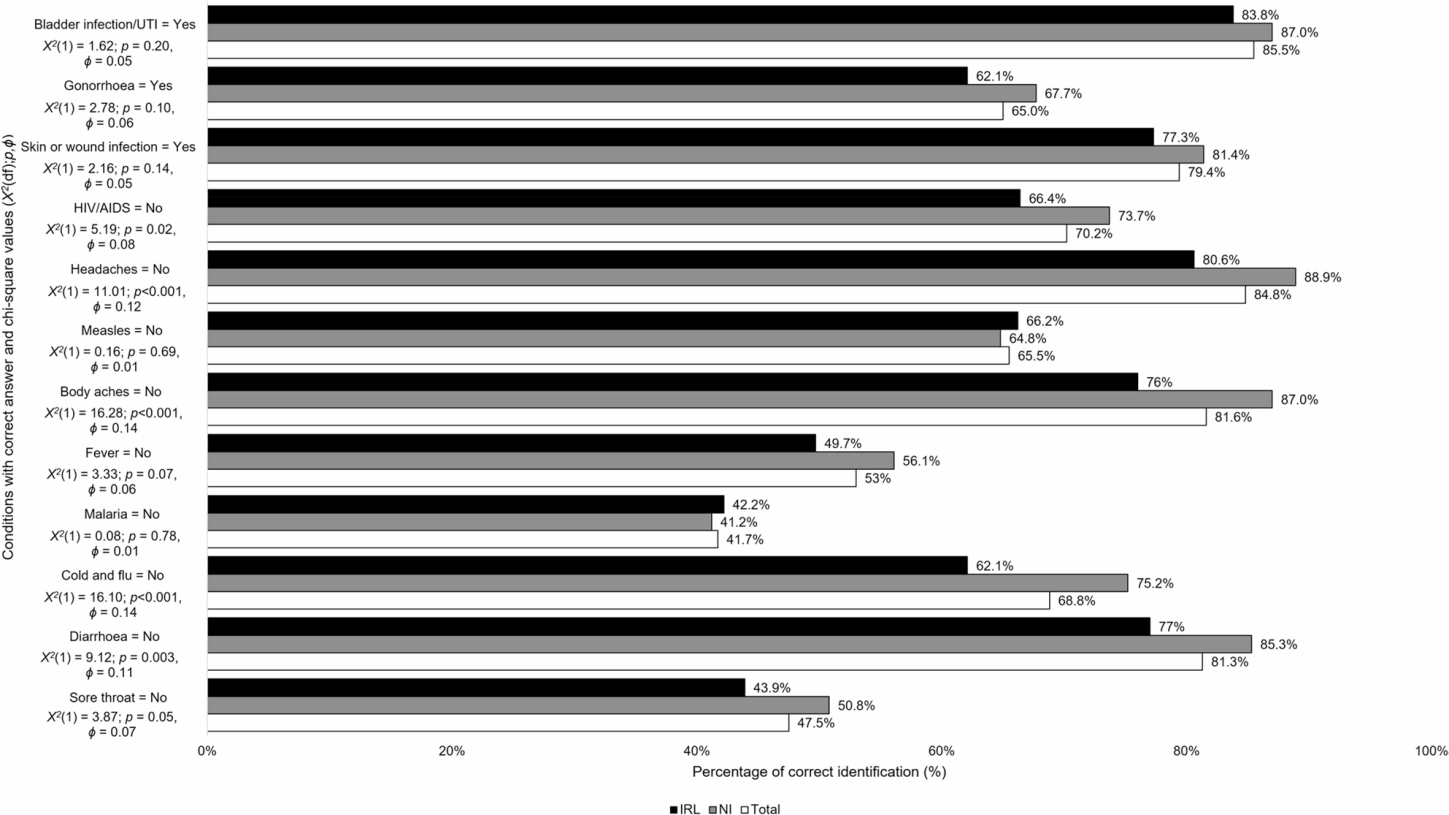
Comparison of IRL and NI respondents correct identification of conditions treatable with antibiotics(*n* = 811)

Average total appropriate antibiotic use knowledge scores (*M*,* SD*) were higher in NI than IRL (10.97, 2.67; 10.08, 2.71 respectively; *p* < 0.001, *d*= 0.33). Those in NI had higher knowledge on when to stop taking antibiotics than those in IRL (*n* = 375,90.4%, *n* = 334,84.3% respectively; *p* = 0.01, *ϕ* = 0.09) and the inappropriateness of using antibiotics based on previous symptoms (*n* = 251, 60.5%, *n* = 206, 52.0% respectively; *p* = 0.02, *ϕ* = 0.09). Knowledge of the inappropriateness of sharing antibiotics was high (*n* = 692, 85.3%) and similar across both countries (*p* = 0.24, *ϕ* = 0.04) (see Table 2). Correct identification of antibiotic-treatable conditions ranged from 41.7% to 85.5% across both countries (see Fig. 1). More people from NI compared to IRL correctly identified sore throat, diarrhoea, cold and flu, body aches, headaches, Human Immunodeficiency Virus/Acquired Immunodeficiency Syndrome (HIV/AIDS) as not being treatable with antibiotics (*p* values ranging from < 0.001 to 0.05, *ϕ* values ranging from 0.07 to 0.14). Cold and flu was incorrectly identified as treatable with antibiotics by nearly a third of the sample (*n* = 558, 31.2%).

**Table 2:** Comparison of IRL and NI respondents’ appropriate antibiotic use knowledge (*n* = 811)

Knowledge items and responses*	M(SD) or *n*(%)			
	Total number (%)	Number in IRL (%)	Number in NI (%)	Χ^2^/t(df);*p*,ϕ/d
‘*When do you think you should stop taking antibiotics?’*				Χ^2^(1)= 6.68;
(1) When you’ve taken all antibiotics as directed	709 (87.4%)	334 (84.3%)	375 (90.4%)	*p* = 0.01, *ϕ* = 0.09
(0) When you feel better/Don’t know	102 (12.6%)	62 (15.7%)	40 (9.6%)	
*‘It’s okay to use antibiotics that were given to a friend or family member, as long as they were used to treat the same illness’*				Χ ^2^(1) = 1.37;
(1) False	692 (85.3%)	332 (83.8%)	360 (86.7%)	*p* = 0.24, *ϕ* = 0.04
(0) True/Don’t know	119 (14.7%)	64 (16.2%)	55 (13.3%)	
*‘It’s okay to buy the same antibiotics, or request these from a doctor, if you’re sick and they helped you get better when you had the same symptoms before’*				Χ ^2^(1) = 5.90;
* (1) False*	457 (56.4%)	206 (52.0%)	251 (60.5%)	*p* = 0.02, *ϕ* = 0.09
(0) True/Don’t know	354 (43.6%)	190 (48.0%)	164 (39.5%)	
Total appropriate antibiotic use knowledge scores	10.53 (2.73)	10.08 (2.71)	10.97 (2.67)	*t*(809) = 4.73; *p* < 0.001,*d* = 0.33

*Correct answer= [1], grouped incorrect answers=(0)

### Knowledge of antibiotic resistance

**Fig. 2:**
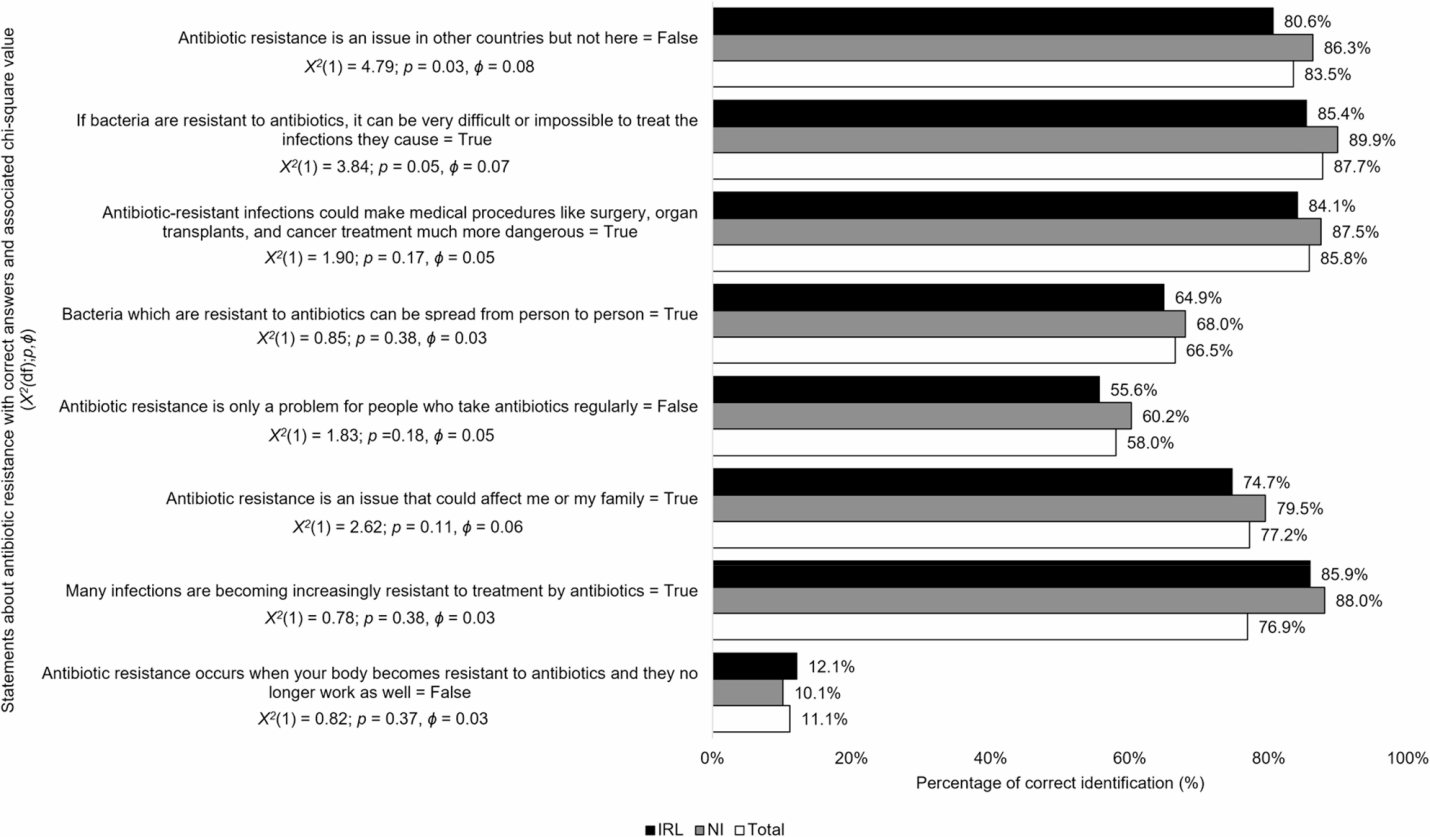
Comparison of IRL and NI survey respondents correct answers on antibiotic resistance facts(*n* = 811)

Those from NI had a higher average total antibiotic resistance knowledge score(*M*,* SD*) compared to IRL (5.69, 1.29; 5.43, 1.4 respectively; (*t*(809) = 2.75; *p* = 0.006, *d* = 0.19)). Those from NI were more knowledgeable on the implications of AMR on treating infections (*p* = 0.05, *ϕ* = 0.07) and that AMR is an issue in their country as well as other countries (*p* = 0.03, *ϕ* = 0.08) compared to those from IRL. Most respondents in both countries incorrectly defined AMR by stating *‘Antibiotic resistance occurs when your body becomes resistant to antibiotics and they no longer work as well”* as true (*n* = 721, 88.9%; *p* = 0.37, *ϕ* = 0.03) (see Fig. 2).

### Knowledge of the use of antibiotics in agriculture

Over a third of respondents (*n* = 289, 35.6%) across both countries said they did not know if antibiotics are widely used in agriculture in Ireland.

### Knowledge of ESKAPE pathogens

**Fig. 3:**
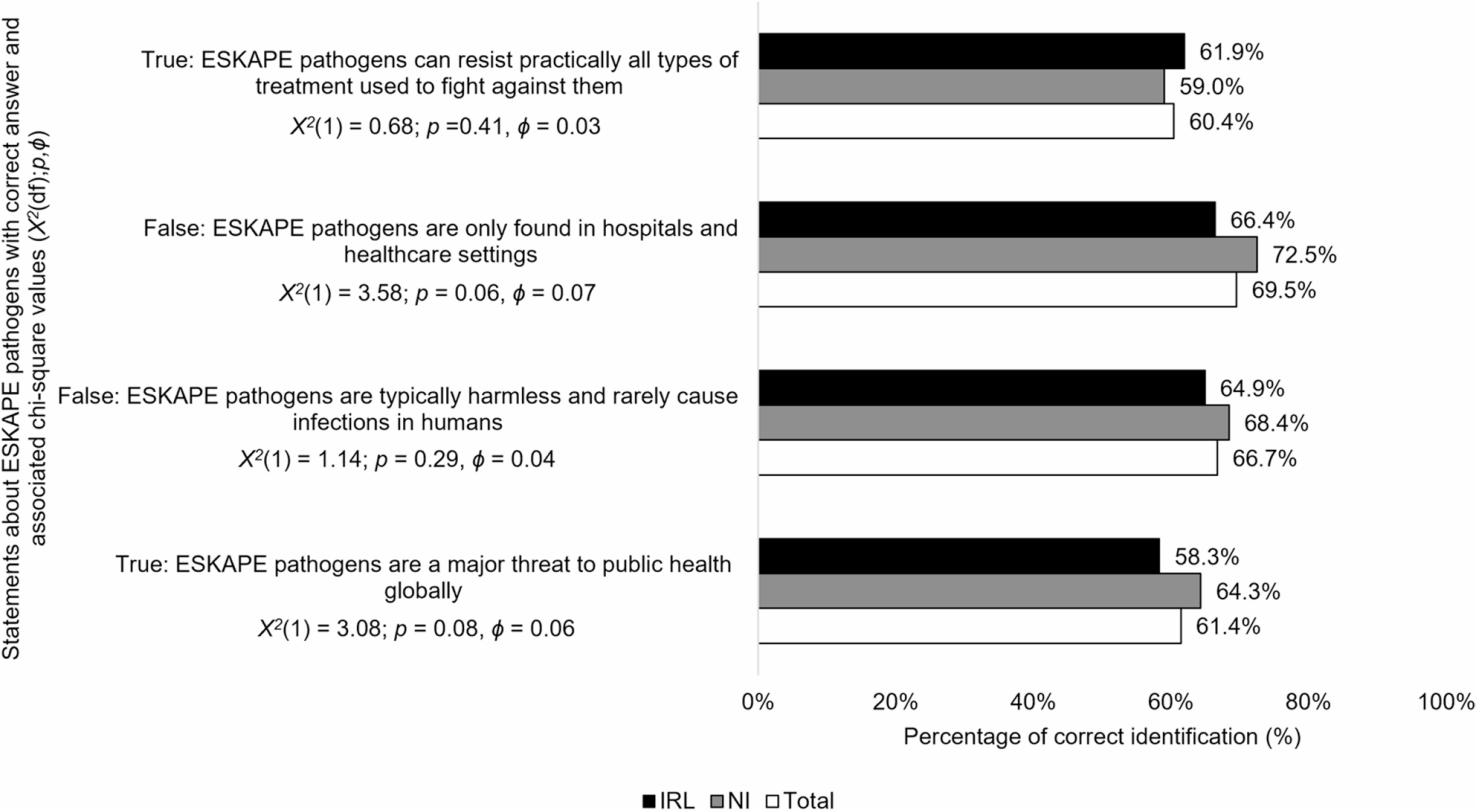
Comparison of IRL and NI survey respondents correct answers on ESKAPE pathogen facts(*n* = 811)

Total ESKAPE pathogen knowledge scores (*M*,* SD*) ranged from 0 to 4 (2.58, 1.07) and were moderate and similar across countries (2.58, 1.07; *t*(809) = 1.71; *p* = 0.09, *d* = 0.12). Around a third to two-fifths of respondents answered the ESKAPE pathogen knowledge questions incorrectly (see Fig. 3).

### Awareness

**Fig. 4:**
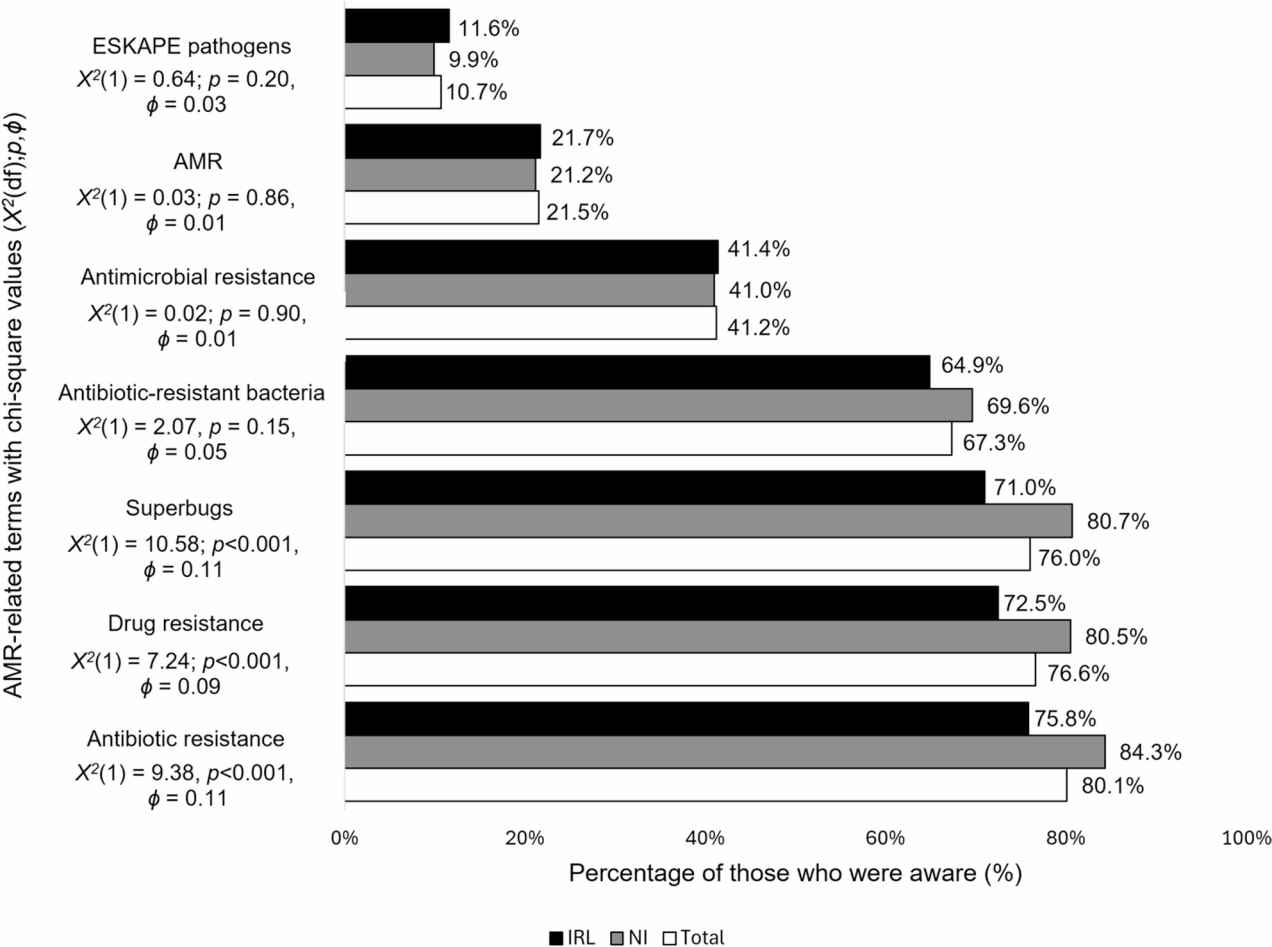
Comparison of NI and IRL survey respondents’ awareness of AMR-related terms(*n* = 811)

**Fig. 5:**
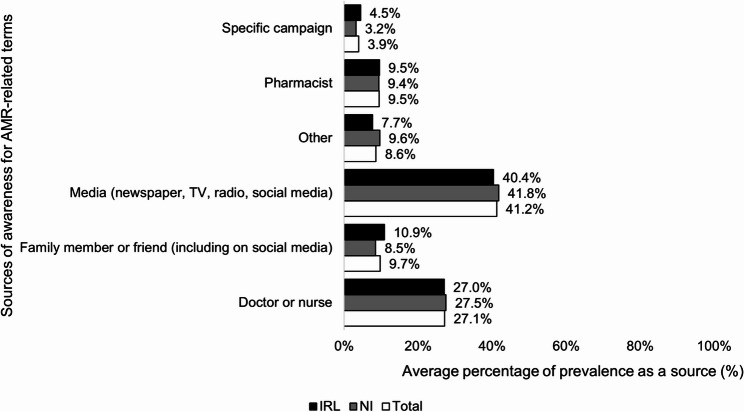
Comparison of NI and IRL survey respondents’ sources of awareness for AMR-related terms(*n* = 159–570)

Awareness(see Fig. 4) was high in both countries for the terms *‘antibiotic resistance’ (*80.1% (*n* = 650), *‘drug resistance’* (76.6% (*n* = 621)) and *‘superbugs’*, (76% (*n* = 616)). More from NI (*n* = 350, 84.3%; *n* = 334, 80.5%, *n* = 335, 80.7% respectively) compared to IRL (*n* = 300, 75.8%; *n* = 287,72.5%; *n* = 281,71% respectively) had heard of them (*p* < 0.001, *ϕ* = 0.11; *p* < 0.001, *ϕ* = 0.09; *p* < 0.001, *ϕ* = 0.11 respectively). Poor levels of awareness were shown across countries of *‘antimicrobial resistance’* (*n* = 334, 41.2%; *p* = 0.90, *ϕ* = 0.01), *‘AMR’* (*n* = 174, 21.5%; *p* = 0.86, *ϕ* = 0.01), and *‘ESKAPE pathogens’* (*n* = 87, 10.7%; *p* = 0.20, *ϕ* = 0.03). Only 6.9% (*n* = 56) had heard of none of the terms. The media (newspaper, television, radio, social media) and a doctor or nurse (34.6%) were the most common sources of awareness in both countries (see Fig. 5).

### Behaviours

Behaviours were consistent across both countries. Over half (57.2%) had taken an antibiotic within the last year (see Table 3).

**Table 3:** Comparison of NI and IRL survey respondents’ most recent antibiotic use behaviours (*n* = 795–811)

Behaviour items and response options	Valid *n*	M(SD) or *n* (%)			
		Total number (%)	IRL number (%)	NI number (%)	Χ^2^(df),*p*,ϕ
Timing of most recent antibiotic use	811				
In the last month		125 (15.4%)	63 (15.9%)	62 (14.9%)	-
In the last 6 months		220 (27.1%)	112 (28.3%)	108 (26.0%)	
In the last year		119 (14.7%)	59 (14.9%)	60 (14.5%)	
More than a year ago		284 (35.0%)	133 (33.6%)	151 (36.4%)	
Never		16 (2.0%)	9 (2.3%)	7 (1.7%)	
Can’t remember		47 (5.8%)	20 (5.1%)	27 (6.5%)	
Antibiotics/prescription were received from doctor/nurse/pharmacist on most recent occasion	795				Χ^2^(1)=0.39;
Yes		740 (93.1%)	358 (92.5%)	382 (93.6%)	*p*=0.53, ϕ = 0.02
No/can’t remember		55 (6.9%)	29 (7.5%)	26 (6.4%)	
Advice on how to take them was received from doctor/nurse/pharmacist on most recent occasion	795				Χ^2^(1)=0.36;
Yes		02 (88.3%)	339 (87.6%)	363 (89%)	*p*=0.55, ϕ = 0.02
No/can’t remember		93 (11.7%)	48 (12.4%)	45 (11.1%)	
Source of obtainment on most recent occasion	795				-
Friend or family member		17 (2.1%)	12 (3.1%)	5 (1.2%)	
Saved up from a previous time		4 (0.5%)	1 (0.3%)	3 (0.7%)	
Medical store or pharmacy		735 (92.5%)	353 (91.2%)	382 (93.6%)	
Somewhere/someone else		5 (0.6%)	1 (0.3%)	4 (1.0%)	
Stall or hawker		4 (0.5%)	3 (0.8%)	1 (0.2%)	
The internet		9 (1.1%)	6 (1.6%)	3 (0.7%)	
Can’t remember		21 (2.6%)	11 (2.8%)	10 (2.5%)	

### Beliefs about ways to address antibiotic resistance

**Fig. 6:**
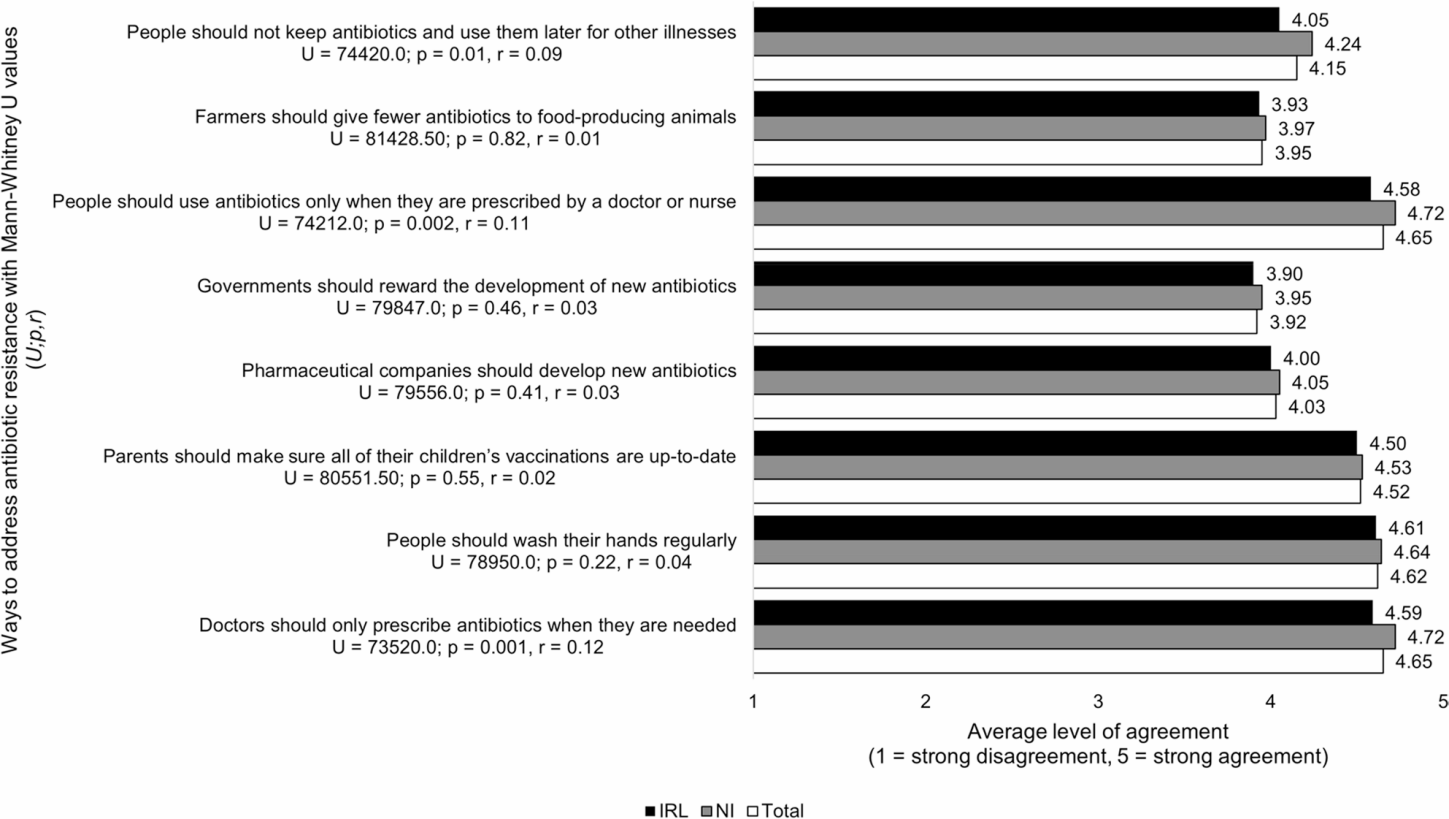
Comparison of NI and IRL survey respondents’ level of agreement with ways to address antibiotic resistance (n = 811)

Those from NI showed higher agreement than those from IRL with the responsible use of antibiotics, for example only using them when prescribed (4.72, 0.65; 4.58, 1.17 respectively; *p* = 0.002, *r* = 0.11) and not keeping them for later illnesses (4.24, 1.09; 4.05, 1.17 respectively; *p* = 0.01, *r* = 0.09). Respondents in both countries strongly believed in handwashing (4.62,0.68; *p* = 0.22,*r* = 0.04) and childhood vaccinations (4.52, 0.84; *p* = 0.55, *r* = 0.02). Uncertainty was evident in both countries around the development of new antibiotics by pharmaceutical companies (4.03, 0.88; *p* = 0.41, *r* = 0.03) and the government rewarding this (3.92,0.99; *p* = 0.46,*r* = 0.03) (see Fig. 6).

### Beliefs about the scale of antibiotic resistance

**Fig. 7:**
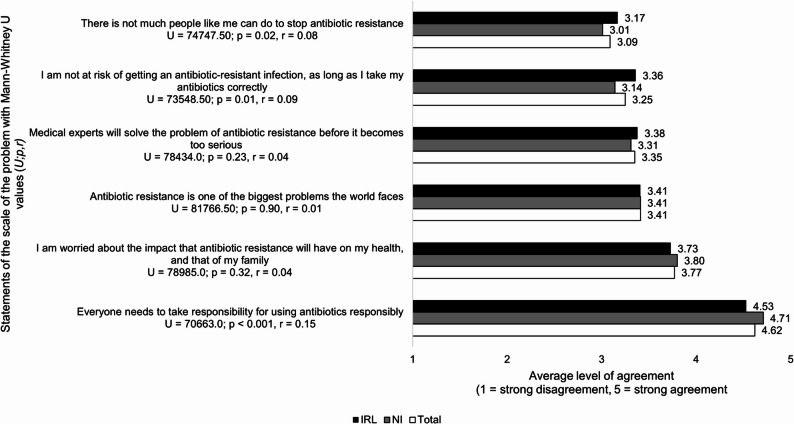
Comparison of NI and IRL survey respondents’ average level of agreement with the scale of antibiotic resistance as a problem (n = 811)

Respondents were neutral, more so in IRL (3.17, 1.04) compared to NI (3.01, 1.13; *p* = 0.02, *r* = 0.08) on believing they can do something to stop AMR. Tentativeness was shown in both countries regarding concern about personal/family health (3.77, 1.03; *p* = 0.32, *r* = 0.04), recognition of antibiotic resistance as a global problem (3.41, 1.08; *p* = 0.90, *r* = 0.01), and confidence in medical experts resolving the issue before it becomes too serious (3.35, 0.95; *p* = 0.23, *r* = 0.04). (see Fig. 7).

### Beliefs about ESKAPE pathogens

**Fig. 8:**
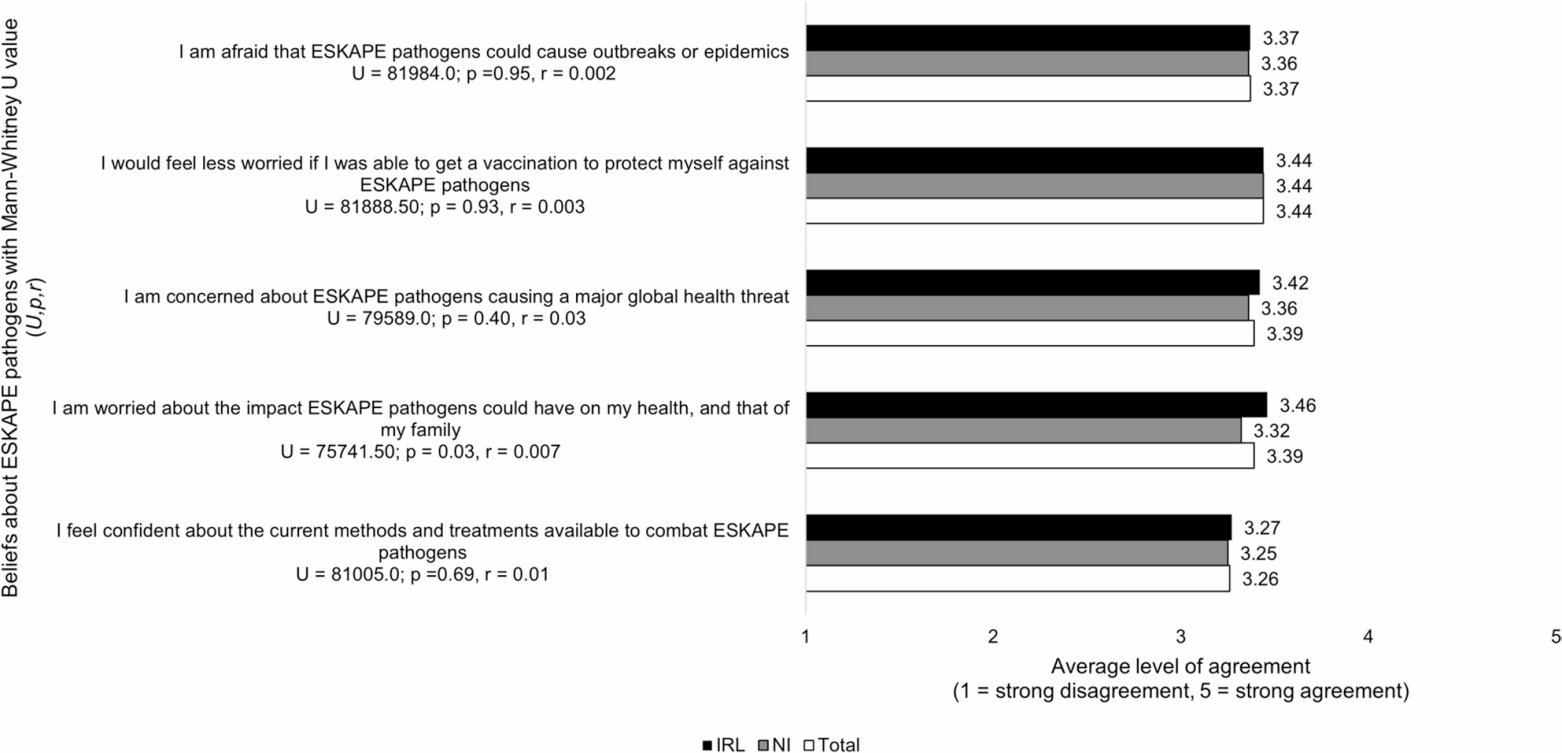
Comparison NI and IRL survey respondents’ average level of agreement with statements about ESKAPE pathogens (*n* = 811)

Respondents in both countries were relatively neutral in their beliefs about ESKAPE pathogens. Those in IRL were more agreeable that they were worried about the impact of ESKAPE pathogens on their/their family’s health compared to those from NI (*p* = 0.03, *r* = 0.07) (see Fig. 8).

## Discussion

This is one of the first cross-border studies on antibiotic and AMR-related knowledge, awareness, beliefs and behaviours across the island of Ireland, and the first in a decade to include an NI sample. Approaches for embedding behavioural science in AMR research has described defining priority behaviours, such as appropriate antimicrobial use and effective infection prevention and control practices, as important for the implementation of NAPs [50]. Overall results suggest those from NI showed higher knowledge and understanding than those from IRL across most topics. However, effect sizes were quite small in most cases, suggesting broad consistency across the island of Ireland. The different community-based initiatives [31, 33] and healthcare systems could play a part in the explanation of differences [28–30], however such inferences are outside the scope of this study. A 2022 analysis of the IRL and NI primary care systems highlighted both are facing similar pressures and perform comparably in population health [51]. In addition, collaborative approaches to tackling AMR are warranted and supported due to the inclusion of co-operation between NI and IRL on health in The Good Friday Agreement (the international peace treaty which outlined a political framework for power-sharing in NI in 1998) [52]. Therefore, a collaborative approach to tackling AMR is justified, as outlined in both countries’ NAPs to addressing AMR [36, 37]. Overall knowledge levels were encouraging in both countries. However, levels of correct identification of antibiotic-treatable conditions were mixed, as well as around two-fifths reported antibiotics can be used based on being prescribed them previously for similar symptoms. These findings may reflect the similarity of viral and bacterial infection symptoms (e.g., sore throat, fever) [53] and pattern recognition from past health experiences influencing subsequent health behaviours [54], rather than poor knowledge. NI and IRL’s current public health messaging encourages people to (i) not share antibiotics and (ii) finish the course as directed. However, the inappropriateness of using antibiotics based on previous symptoms, or the overlap of viral and bacterial symptoms, are not explicitly mentioned [55, 56]. Public health messaging should strive to cover all these elements to reinforce appropriate antibiotic use. Decision support and risk stratification tools, such as correct diagnosis and optimal drug, are highlighted as useful aids for clinicians to ensure prescriptions are appropriate in the case of viral vs. bacterial infections [37]. These practices could help reduce the potential incidence of inappropriate antibiotic consumption for viral infections across the island of Ireland. It is a particular concern that cold and flu (common viral infections) were incorrectly identified by nearly a third of our sample as being antibiotic-treatable. Notably, neither are included in pharmacy-based services which aim to support the management of common conditions [31, 32]. An evaluation of one such service for sore throat showed 81% of service users said they felt confident to manage symptoms themselves in the future, and GPs felt the service reduced inappropriate prescribing of antibiotics and provided an opportunity to provide patient education about self-care [57]. As a result, broadening the remit of these services could benefit from inclusion of the cold and flu, to improve patient understanding and reduce the potential inappropriate use of antibiotics. In addition, a community-based initiative has shown improvements to participants’ health literacy, including following pharmacist instructions and finding treatment information [58]. The success of such initiatives suggest that programmes delivered locally could provide a platform for engaging and educating the public on issues like appropriate antibiotic use and AMR; an objective in both countries’ NAPs [36, 59]. It could therefore be strategic to explore the creation and implementation of community-based AMR initiatives in both countries. The media and a doctor or nurse were the most cited sources of AMR awareness in both countries. A review of the effectiveness of AMR-related campaigns found TV advertising and healthcare professional-patient interaction as recurring features of those which improved knowledge and awareness outcomes [60]; this could explain the moderate levels exhibited in this study. Previous UK awareness-raising campaigns used single-channel approaches (posters/leaflets) and evaluations emphasised the need for multi-media, integrated social marketing and communications campaigns [61]. Current campaigns across the island of Ireland currently use both mediums [34, 35] and should continue to do so as a result. With that said, the reach of the media, particularly social media platforms, could be problematic due to its potential to provide unreliable and misleading information [62–65]. Both countries’ NAPs commit to using educational settings and engagement guides to provide the public with consistent and reliable AMR-related messaging [36, 59]. The content of such should include guidance on evaluating AMR-related information obtained via the media. Moderate knowledge and uncertainty was exhibited around ESKAPE pathogens. No reference to ESKAPE pathogens could be found in the NI and IRL AMR awareness raising campaigns [34, 35] which could explain these findings. ESKAPE pathogens are part of the WHO’s bacterial priority pathogens list [66], and a recent 2024 review on ESKAPE pathogens calls for high-income countries (like NI and IRL) to commit to strong infection prevention strategies and ensuring the appropriate use of antibiotics [67]. As a result, public health messaging across the island of Ireland should begin to reference ESKAPE pathogens to increase awareness and promote positive behaviours which mitigate their threat. This would be particularly beneficial in clinical environments due to ESKAPE pathogens’ implications in life-threatening hospital infections [66, 68]. Similar to global research, awareness was low in both countries for more technical and abbreviated terms like ‘antimicrobial resistance’, ‘AMR’ [10], and ‘ESKAPE pathogens’. Such terms have shown to perform poorly in relation to memorability and risk perception and are described as unsuitable for health communication [69]. Research has shown that expansion of medical abbreviations can improve patient understanding of health information [70] and conceptual change theory [71] explains that misconceptions of basic definitions can impede overall learning and understanding of a topic. As a result, future health communications in both countries should refrain from using acronyms and continue to utilise terms like ‘antibiotic resistance’ and ‘drug resistance’, to effectively engage the public [72, 73]. Appropriate antibiotic obtainment behaviour results were encouraging as these reduce the likelihood of inappropriate antibiotic use which is a main driver of AMR [11, 12]. However, over half (57%) of people self-reported antibiotic use within the last year. While timing of use does not mean such use is inappropriate, these levels are still a concern due to more use increases the likelihood of bacteria becoming resistant and escalating the prevalence of AMR [11, 12]. These findings support both countries’ NAPs commitment to the continual monitoring of antibiotics and promotion of appropriate prescribing [36, 37, 59, 74] in an aim to reduce consumption and ensure consumption only occurs when necessary to reduce the risk of AMR. There was a gap in understanding of the use of antibiotics in agriculture and showed agreement that medical experts will solve the problem of AMR before it becomes too serious. Support for a cross-sectoral and unified approach to tackling AMR has been widely documented [6, 15, 75]. Although both NI and IRL’s NAPs explicitly reference its importance [36, 37], these results show a potential for the ‘non-accountability effect’, which can enable the diffusion of personal responsibility and could elicit inaction by the public in addressing AMR [76]. Qualitative research with agriculture stakeholders shows simply aiming to fill the knowledge gap in this area is not productive, and instead suggests the use of stories focusing on shared values and emotions (e.g., animal welfare) [77]. Other research suggests community-based initiatives, such as workshops and outreach from stakeholders and community leaders, can empower individuals to recognise their role in combatting AMR [78]. Our findings echo the need for multiple communication efforts that target and leverage the values and belief systems of different consumer groups to reinforce the shared responsibility in tackling AMR [79]. There was a strong belief in both countries in parents making sure their children’s vaccinations are up to date. This suggests that if vaccines targeting AMR bacteria became available, the public across the island of Ireland may engage in their uptake. However, recent statistics (2022/23) show childhood vaccine uptake rates in both countries are below the 95% WHO coverage target. For example, MMR vaccine uptake was 89.8% (Health Service Executive (HSE)-administered) and 80.4% (GP-administered) in IRL, and fell from 94.9% to 93.6% in NI in the same period [80–82]. Given the widely documented role of vaccines in reducing the burden of AMR [83, 84], efforts to sustain and modestly improve vaccine uptake rates could be beneficial to ensure potential new AMR vaccines optimise their protective impact. This study’s findings improve understanding of cross-border behavioural dynamics related to AMR across the island of Ireland, of which are objectives in both NI and IRL’s NAPs [36, 59]. It is one of the first studies to attempt to assess knowledge and beliefs related to ESKAPE pathogens as no standardised measure could be found, showing potential knowledge gaps and uncertainty. While the sample was nationally representative based on the most recent NI and IRL national Census data on sex [43, 44], there was a mixed representation of certain subgroups (e.g., 7.4% more 35–44 year olds in IRL compared to NI), potentially limiting the generalisability of the findings to certain sub-groups. Diagnosing the barriers and enablers of AMR-related behaviours, including cultural and social factors, is necessary to design effective interventions [50]. Recent IRL and NI qualitative research shows such factors; e.g., caring responsibilities, differing experiences with healthcare professionals, can influence misuse of antibiotics, such as not finishing a course or using them to relieve symptoms [65, 85]. Qualitative methods have been highlighted as helpful in exploring these factors and informing the development of behavioural AMS interventions [86]. Future research could use these methods to build on this study’s findings so that interventions can be tailored for diverse sub groups and settings across both countries.

## Conclusion

To the authors’ knowledge, this study provides a novel understanding of knowledge, awareness, behaviours and beliefs around antibiotics and AMR across the island of Ireland. Despite findings being mostly encouraging for appropriate antibiotic use and effective infection prevention practices, gaps were found including identifying conditions that require antibiotics, understanding ESKAPE pathogens, and recognising personal responsibility in addressing AMR. Community and pharmacy-based initiatives could be used to educate the public on AMR and responsible antibiotic use. Campaigns and messaging should refrain from using acronyms and employ multi-channel strategies to foster shared responsibility and encourage positive AMR-related behaviours across the island of Ireland.

## Supplementary Information

Supplementary Material 1.

## Data Availability

Participant privacy prevents the public sharing of data. Analysis output files can be shared by the corresponding author on request. This manuscript complies with The Strengthening the Reporting of Observational Studies in Epidemiology (STROBE) statement for cross-sectional studies ( https:/doi.org/10.1016/j.jclinepi.2007.11.008 ). The study was registered to use the EQ-5D-3 L (registration ID 66281).

## Abbreviations

AIDS: Acquired Immunodeficiency Syndrome
AIVRT: All-Island Vaccine Research and Training Alliance
AMR: Antimicrobial resistance
AMS: Antimicrobial stewardship
COVID-19: Coronavirus Disease 2019
EEA: European Economic Area
EQ-5D-3L: EuroQol-5 Dimensions-3 Levels
EQ-VAS: EuroQol-Visual Analogue Scale
ESKAPE: Enterococcus faecium, Staphylococcus aureus, Klebsiella pneumoniae, Acinetobacter baumannii, Pseudomonas aeruginosa, and Enterobacter species
EU: European Union
GP: General Practitioner
HEA: Higher Education Authority
HIV: Human Immunodeficiency Virus
HSE: Health Service Executive
IBM®: International Business Machines®
IRL: Ireland
M, SD: Mean, Standard Deviation
NAPs: National Action Plans
NI: Northern Ireland
NSRP: North South Research Programme
SPSS®: Statistical Package for the Social Sciences®
STROBE: The Strengthening the Reporting of Observational Studies in Epidemiology
UTI: Urinary Tract Infection
WHO: World Health Organization
