# Correction: Cross-border differences in public knowledge, awareness, behaviours and beliefs related to antibiotics and antimicrobial resistance across the island of Ireland

**DOI:** 10.1186/s12889-026-27802-6

**Published:** 2026-05-27

**Authors:** Caoimhe Shields, Emma Berry, Laura J. Sahm, Aoife Fleming, Chikondi C. Kandulu, Anne C. Moore, Gillian W. Shorter

**Affiliations:** 1 School of Psychology, Queen’s University Belfast, Belfast, Northern Ireland; 2 Pharmaceutical Care Research Group, School of Pharmacy, University College Cork, Cork, Ireland; 3 School of Biochemistry and Cell Biology, University College Cork, Cork, Ireland; 4 National Institute for Bioprocessing Research and Training, Dublin, Ireland; 5 SSPC Pharmaceutical Research Centre, University College Cork, Cork, Ireland; 6 School of Nursing and Midwifery, Trinity College Dublin, The University of Dublin, Dublin, Ireland; 7 TreAdd Research Group on Treatment and Addictions, Tampere University, Tampere, Finland; 8 Queen’s University Belfast, University Road, Belfast, BT7 1NN Northern Ireland

**Correction: BMC Public Health 26**,** 1327 (2026)**

**https://doi.org/10.1186/s12889-026-27024-w**

Following publication of the original article [1], the authors reported an error in PDF version of the published article. The phi symbol “*ϕ*” displaying in the PDF version of the published article is incorrect, while the html version of the article has no issue.

The incorrect symbols are found in the paragraphs under the following headings: “Knowledge of appropriate antibiotic use”, “Knowledge of antibiotic resistance”, “Awareness” and in Table 2.

The original article [1] has been updated.
