# Supplementary material for: Cross-border differences in public knowledge, awareness, behaviours and beliefs related to antibiotics and antimicrobial resistance across the island of Ireland
Source: BMC Public Health. 2026 Mar 16;26:1327. doi: 10.1186/s12889-026-27024-w (PMC13104443; doi:10.1186/s12889-026-27024-w)
Supplement: Supplementary file 1 — Supplementary Material 1. [file 12889_2026_27024_MOESM1_ESM.docx]

**Supplementary material 1: Full survey questionnaire**

**DEMOGRAPHICS**

**D1 What gender do you best identify with?**

Male

Female

Prefer to self-describe

Prefer not to say

**D2 What age are you?**

Open box to enter age in years

**D3 In which of these countries do you currently live?**

Northern Ireland

Republic of Ireland

**D4 Which of these best describes where you live?**

Urban – within a densely populated city/town

Suburban – in a suburb of a city/town

Rural – outside of a city/town, e.g. village/countryside/farming area

**D5 What is the highest level of education attainment you have received?**

*If currently enrolled, please select the highest degree you have already received.*

No qualifications

Essential skills / Level 3 Junior Certificate

GCSE / Level 4 Leaving Certificate or similar

AS / A Level / Level 5 Leaving Certificate or similar

Technical qualification

Bachelor’s degree

Master’s/Professional degree

Doctorate degree

**D6 Which of following best describes your total household income, before tax per year?**

Less than £/€15,000

£/€15,000 - £/€29,999

£/€30,000 - £/€44,999

£/€45,000 - £/€59,999

£/€60,000 or more

**D7 What is your ethnicity?**

Bangladeshi

Chinese

Indian

Pakistani

Asian Other

Black African

Black Caribbean

Black Other

Mixed ethnic group

White

White Irish Traveller

White Roma

White Other

Other ethnic group

**D8 Which of these best describes your household composition?**

1 single adult only

1 single adult and at least 1 child under 16

Married / domestic partnership - adults only

Married / domestic partnership and at least 1 child under 16

Multiple adults aged 16+ only

Multiple adults aged 16+ and at least 1 child under 16

**D9 Do you work in any of the following sectors?**

- Healthcare
- Public Health
- Veterinary Medicine
- Pharmaceutical Industry
- Agriculture
- Environmental Science
- Policy and Government
- Research and Academia

Yes

No

**D10 Under each heading, please tick the ONE box that best describes your health TODAY.**

***MOBILITY***

I have no problems in walking about

I have some problems in walking about

I am confined to bed

***SELF-CARE***

I have no problems with self-care

I have some problems washing or dressing myself

I am unable to wash or dress myself

***USUAL ACTIVITIES (e.g. work, study, housework, family or leisure activities)***

I have no problems with performing my usual activities

I have some problems with performing my usual activities

I am unable to perform my usual activities

***PAIN / DISCOMFORT***

I have no pain or discomfort

I have moderate pain or discomfort

I have extreme pain or discomfort

***ANXIETY / DEPRESSION***

I am not anxious or depressed

I am moderately anxious or depressed

I am extremely anxious or depressed

**D11** **How good or bad is your health TODAY? (0 means the worst health you can imagine, 100 means the best health you can imagine)**

Response box to enter number/scale to place number

**MAIN QUESTIONNAIRE**

**SECTION 1: USE OF ANTIBIOTICS**

**1) When did you last take antibiotics?**

In the last month

In the last 6 months

In the last year

More than a year ago

Never

Can’t remember

If ‘Never’ code 5, go straight to Question 5.

**2) On that occasion, did you get the antibiotics (or a prescription for them) from a doctor, nurse or pharmacist?**

Yes

No

Can’t remember

**3) On that occasion, did you get advice from a doctor, nurse or pharmacist on how to take them?**

Yes, I received advice on how to take them (e.g. with food, for 7 days)

No

Can’t remember

**4) On that occasion, where did you get the antibiotics?**

Medical store or pharmacy

Stall or hawker

The internet

Friend or family member

I had them saved up from a previous time

Somewhere/someone else

Can’t remember

**SECTION 2:** **KNOWLEDGE ABOUT ANTIBIOTICS**

**5) When do you think you should you stop taking antibiotics once you’ve begun treatment?**

When you feel better

When you’ve taken all of the antibiotics as directed

Don’t know

**6) Do you think this statement is ‘true’ or ‘false’?**

***“It’s okay to use antibiotics that were given to a friend or family member, as long as they were used to treat the same illness”***

True

False

Don’t know

**7) Do you think this statement is ‘true’ or ‘false’?**

***“It’s okay to buy the same antibiotics, or request these from a doctor, if you’re sick and they helped you get better when you had the same symptoms before”***

True

False

Don’t know

**8) Do you think these conditions can be treated with antibiotics?**

Sore throat

Diarrhoea

Cold and flu

Skin or wound infection

Malaria

Fever

Gonorrhoea

Body aches

Measles

Bladder infection or urinary tract infection (UTI)

Headaches

HIV/AIDS

**SECTION 3: KNOWLEDGE ABOUT ANTIBIOTIC RESISTANCE**

**9) Have you heard of any of the following terms?**

Antibiotic resistance

Superbugs

Antimicrobial resistance

AMR

Drug resistance

Antibiotic-resistant bacteria

Yes

No

**10)**

***10_1. Ask if answered YES @ 9) to ‘Antibiotic Resistance’***

**Where did you hear about the term: ‘Antibiotic Resistance’?**

Doctor or nurse

Pharmacist

Family member or friend (including on social media)

Media (newspaper, TV, radio, social media)

Specific campaign

Other

Can’t remember

***10_2. Ask if answered YES @ 9) to ‘Superbugs’***

**Where did you hear about the term: ‘Superbugs’?**

Doctor or nurse

Pharmacist

Family member or friend (including on social media)

Media (newspaper, TV, radio, social media)

Specific campaign

Other

Can’t remember

***10_3. Ask if answered YES @ 9) to ‘Antimicrobial Resistance’***

**Where did you hear about the term: ‘Antimicrobial resistance’?**

Doctor or nurse

Pharmacist

Family member or friend (including on social media)

Media (newspaper, TV, radio, social media)

Specific campaign

Other

Can’t remember

***10_4. Ask if answered YES @ 9) to ‘AMR’***

**Where did you hear about the term: ‘AMR’?**

Doctor or nurse

Pharmacist

Family member or friend (including on social media)

Media (newspaper, TV, radio, social media)

Specific campaign

Other

Can’t remember

***10_5. Ask if answered YES @ 9) to ‘Drug resistance’***

**Where did you hear about the term: ‘Drug resistance’?**

Doctor or nurse

Pharmacist

Family member or friend (including on social media)

Media (newspaper, TV, radio, social media)

Specific campaign

Other

Can’t remember

***10_6. Ask if answered YES @ 9) to ‘Antibiotic-resistant bacteria’***

**Where did you hear about the term: ‘Antibiotic-resistant bacteria’?**

Doctor or nurse

Pharmacist

Family member or friend (including on social media)

Media (newspaper, TV, radio, social media)

Specific campaign

Other

Can’t remember

**11) Please indicate whether you think the following statements are ‘true’ or ‘false’**

Antibiotic resistance occurs when your body becomes resistant to antibiotics and they no longer work as well.

Many infections are becoming increasingly resistant to treatment by antibiotics.

If bacteria are resistant to antibiotics, it can be very difficult or impossible to treat the infections they cause.

Antibiotic resistance is an issue that could affect me or my family.

Antibiotic resistance is an issue in other countries but not here.

Antibiotic resistance is only a problem for people who take antibiotics regularly.

Bacteria which are resistant to antibiotics can be spread from person to person.

Antibiotic-resistant infections could make medical procedures like surgery, organ transplants, and cancer treatment much more dangerous.

**12) On the scale shown, how much do you agree the following actions would help address the problem of antibiotic resistance?**

|  | | Strongly agree | Agree | Neither agree nor disagree | Disagree | Strongly disagree |
| --- | --- | --- | --- | --- | --- | --- |
| 1 | Doctors should only prescribe antibiotics when they are needed. | 5 | 4 | 3 | 2 | 1 |
| 2 | People should wash their hands regularly. | 5 | 4 | 3 | 2 | 1 |
| 3 | Parents should make sure all of their children’s vaccinations are up-to-date. | 5 | 4 | 3 | 2 | 1 |
| 4 | Pharmaceutical companies should develop new antibiotics. | 5 | 4 | 3 | 2 | 1 |
| 5 | Governments should reward the development of new antibiotics. | 5 | 4 | 3 | 2 | 1 |
| 6 | People should use antibiotics only when they are prescribed by a doctor or nurse. | 5 | 4 | 3 | 2 | 1 |
| 7 | Farmers should give fewer antibiotics to food-producing animals. | 5 | 4 | 3 | 2 | 1 |
| 8 | People should not keep antibiotics and use them later for other illnesses. | 5 | 4 | 3 | 2 | 1 |

**13) On the scale shown, how much do you agree with following statements?**

|  | | Strongly agree | Agree | Neither agree nor disagree | Disagree | Strongly disagree |
| --- | --- | --- | --- | --- | --- | --- |
| 1 | Everyone needs to take responsibility for using antibiotics responsibly. | 5 | 4 | 3 | 2 | 1 |
| 2 | I am worried about the impact that antibiotic resistance will have on my health, and that of my family. | 5 | 4 | 3 | 2 | 1 |
| 3 | Antibiotic resistance is one of the biggest problems the world faces. | 5 | 4 | 3 | 2 | 1 |
| 4 | Medical experts will solve the problem of antibiotic resistance before it becomes too serious. | 5 | 4 | 3 | 2 | 1 |
| 5 | I am not at risk of getting an antibiotic-resistant infection, as long as I take my antibiotics correctly. | 5 | 4 | 3 | 2 | 1 |
| 6 | There is not much people like me can do to stop antibiotic resistance. | 5 | 4 | 3 | 2 | 1 |

**SECTION 4: USE OF ANTIBIOTICS IN AGRICULTURE**

**14) Do you think antibiotics are widely used in agriculture (including in food-producing animals) in your region?**

Yes

No

Don’t know

***SECTION 5: KNOWLEDGE ABOUT ESKAPE PATHOGENS***

***15) Have you heard of the term ‘ESKAPE pathogens’?***

Yes

No

***15_1. Ask if answered YES @ 15) to ‘ESKAPE pathogens’***

**Where did you hear about the term: ‘ESKAPE pathogens’?**

Doctor or nurse

Pharmacist

Family member or friend (including on social media)

Media (newspaper, TV, radio)

Specific campaign

Other

Can’t remember

**16) Please indicate whether you think the following statements are ‘true’ or ‘false’**

ESKAPE pathogens are a major threat to public health globally

ESKAPE pathogens are typically harmless and rarely cause infections in humans

ESKAPE pathogens are only found in hospitals and healthcare settings

ESKAPE pathogens can resist practically all types of treatment used to fight against them

**17) On the scale shown, how much do you agree with the following statements about ESKAPE pathogens?**

|  | | Strongly agree | Agree | Neither agree nor disagree | Disagree | Strongly disagree |
| --- | --- | --- | --- | --- | --- | --- |
| 1 | I feel confident about the current methods and treatments available to combat ESKAPE pathogens | 5 | 4 | 3 | 2 | 1 |
| 2 | I am worried about the impact ESKAPE pathogens could have on my health, and that of my family | 5 | 4 | 3 | 2 | 1 |
| 3 | I am concerned about ESKAPE pathogens causing a major global health threat | 5 | 4 | 3 | 2 | 1 |
| 4 | I would feel less worried if I was able to get a vaccination to protect myself against ESKAPE pathogens | 5 | 4 | 3 | 2 | 1 |
| 5 | I am afraid that ESKAPE pathogens could cause outbreaks or epidemics | 5 | 4 | 3 | 2 | 1 |

**18) Is there anything else you would like to tell us about your thoughts on antimicrobial resistance?**

Response box for open-ended responses.

**SECTION 6: KNOWLEDGE ABOUT VACCINES**

**19) On the scale shown, how much do you agree with each of the following statements about vaccination?**

|  | | Strongly agree | Agree | Neither agree nor disagree | Disagree | Strongly disagree |
| --- | --- | --- | --- | --- | --- | --- |
| 1 | Vaccines are important for health | 5 | 4 | 3 | 2 | 1 |
| 2 | Vaccines are effective | 5 | 4 | 3 | 2 | 1 |
| 3 | Getting vaccinated is important for the health of others in my community | 5 | 4 | 3 | 2 | 1 |
| 4 | All vaccines offered by the government program in my community are beneficial | 5 | 4 | 3 | 2 | 1 |
| 5 | New vaccines carry more risks than older vaccines | 5 | 4 | 3 | 2 | 1 |
| 6 | The information I receive about vaccines from vaccine programs is reliable and trustworthy | 5 | 4 | 3 | 2 | 1 |
| 7 | Getting vaccines is a good way to protect from disease | 5 | 4 | 3 | 2 | 1 |
| 8 | Generally I do what my doctor or health care provider recommends about vaccines | 5 | 4 | 3 | 2 | 1 |
| 9 | I am concerned about serious adverse effects of vaccines | 5 | 4 | 3 | 2 | 1 |
| 10 | I do not need vaccines for diseases that are not common anymore | 5 | 4 | 3 | 2 | 1 |
| 11 | Vaccines are important for children to have | 5 | 4 | 3 | 2 | 1 |
| 12 | Vaccines are safe | 5 | 4 | 3 | 2 | 1 |
| 13 | Vaccines are compatible with my religious, personal or philosophical beliefs | 5 | 4 | 3 | 2 | 1 |

**20)** If you have anything further to say about the topics covered in this survey, please write it below

Open ended response box

**END OF QUESTIONNAIRE**
